# Perioperative management of patients with antiphospholipid and catastrophic antiphospholipid syndrome undergoing urgent neurosurgery

**DOI:** 10.3389/fphar.2023.1253655

**Published:** 2023-09-01

**Authors:** Knarik Ginosyan, Hasmik Misakyan, Arman Zakaryan

**Affiliations:** ^1^ Department of Rheumatology, Yerevan State Medical University After Mkhitar Heratsi, Yerevan, Armenia; ^2^ Department of Surgery, ENT Division, “ArtMed” RMC, Yerevan, Armenia; ^3^ Department of Surgery, Neurosurgery Division, “ArtMed” RMC, Yerevan, Armenia

**Keywords:** urgent neurosurgery, antiphospholipid syndrome, catastrophic antiphospholipid syndrome, perioperative management, plasmapheresis

Acute neurosurgical conditions associated with brain compression, swelling, dislocation, and herniation require emergency surgery. These time-sensitive and life-saving procedures aim to reverse or stop further damage to the central nervous system, the success of which depends on immediate surgery. Despite this urgency, any co-morbidities, which may complicate the perioperative course, should be taken into account while developing a treatment plan. Patients with antiphospholipid syndrome (APS) and catastrophic antiphospholipid syndrome (CAPS) receiving anticoagulation therapy have an increased risk of perioperative thrombotic/bleeding complications. Perioperative management of such cases is a complex problem; moreover, these co-morbidities themselves may cause a neurosurgical emergency. Guidelines and studies for the perioperative management of pathologies with high thrombotic/bleeding risk recommend that patients undergoing a neurosurgical procedure should discontinue therapy with oral anticoagulants before surgery and continue therapy with heparin or low molecular weight heparin (LMWH) (Zakaryan, 2014; Douketis et al., 2022; Shah et al., 2023). However, such a scheme is applicable in cases with planned neurosurgical operations. In neurosurgical emergencies, additional strategies for managing the perioperative course are needed. This brief communication summarizes currently available approaches to perioperative management of patients with APS and CAPS undergoing urgent neurosurgical procedures.

APS is a systemic autoimmune disorder characterized by venous or arterial thrombosis in the presence of antiphospholipid antibodies: anticardiolipin antibodies IgG/IgM, anti-β2-glycoprotein-I antibodies IgG/IgM, lupus anticoagulant. APS may occur as a primary condition but also develop in the presence of systemic lupus erythematosus and other systemic autoimmune diseases (Tektonidou et al., 2019; Rodziewicz and D'Cruz, 2020). Patients with APS should receive long-term treatment with vitamin K antagonists along with low-dose aspirin when indicated. In case of recurrent thrombosis, the patients may need LMWH (Finazzi et al., 2005; Ruiz-Irastorza et al., 2011; Tektonidou et al., 2019). CAPS is a rare APS variant characterized by multiple small vessel thromboses, leading to multi-organ failure and insufficiency. Neurosurgical complications occur in one-third of CAPS patients (Drazin et al., 2014). In rare cases, CAPS emerges with cerebellar bleeding requiring urgent neurosurgery (Drazin et al., 2014). Several APS/CAPS-associated neurosurgical cases are described in the searchable literature (Inoue et al., 1994; Nagai et al., 1998; Miesbach et al., 2004; Cervera et al., 2005; Finis et al., 2005; Arinuma et al., 2011; Drazin et al., 2014; Arias et al., 2019). Among these reported cases best outcomes were observed in patients managed with plasmapheresis perioperatively (Miesbach et al., 2004; Drazin et al., 2014; Arias et al., 2019). Miesbach et al. (2004) reported a case of CAPS-related bilateral subdural hematoma treated with plasmapheresis before neurosurgery (left frontal craniotomy, right frontal drill-hole trepanation with drainage tubes) with minimal postoperative neurological deficit and good outcome at discharge. Drazin et al. (2014) presented a unique case with CAPS-related cerebellar hematoma, idiopathic thrombocytopenic purpura, deep vein thrombosis, infarctions in the kidneys and spleen, adrenal hemorrhage, and altered mental status. The patient acutely deteriorated secondary to the development of a cerebellar subdural hematoma requiring an emergent decompression and excision of the hematoma. After recovery in the intensive care unit, the patient developed a new spontaneous epidural hematoma necessitating an additional surgical intervention. The patient received six courses of plasmapheresis which made it possible to decrease the level of antiphospholipid antibodies creating the possibility to conduct the neurosurgical procedure. Arias et al. (2019) reported two cases of APS when patients successfully received perioperative plasmapheresis for performing an extracranial-intracranial bypass (ECIC) to treat a left internal carotid artery aneurysm in one case and moyamoya disease in the second patient. Both cases were managed with perioperative plasmapheresis to avoid the need for anticoagulation during the perioperative period, and both patients underwent successful ECIC bypass procedures without perioperative ischemic or hemorrhagic complications.

Plasmapheresis or therapeutic plasma exchange is used to remove autoantibodies, immune complexes, cytokines, and pathologic inflammatory mediators from the circulation. It can be used in the perioperative period in autoimmune diseases (Roman et al., 2014; Prouvot et al., 2019; Rodriguez-Pinto et al., 2019). Fresh frozen plasma (FFP) is the most commonly used plasma product to correct clotting factor deficiencies, and its use could potentially reduce the bleeding risk in these patients. Along with FFP, cryoprecipitate, and recombinant factor concentrates are used as an option before neurosurgery in coagulopathic patients.

In conclusion, plasmapheresis and/or FFP along with cryoprecipitate and recombinant factor concentrates may be used for the management of APS and CAPS in urgent neurosurgical cases perioperatively. However, it is still unclear if their use could worsen the thrombotic storm of CAPS (Marson et al., 2008). It might be reserved for a subgroup of patients at higher risk of bleeding. Further multicenter trials are needed to estimate the safety, effectiveness, and limitations of this method in emergency neurosurgery.
